# Functional role of microRNA-135a in colitis

**DOI:** 10.1186/s12950-018-0181-z

**Published:** 2018-04-06

**Authors:** Chunyan Lou, Yanyang Li

**Affiliations:** Department of Pediatrics, Huaihe Hospital of Henan University, No. 8, Baobei Road, Gulou District, Kaifeng, 475000 China

**Keywords:** Murine model of DSS-induced colitis, miR-135a, Hif1α, Inflammatory bowel disease

## Abstract

**Background:**

Inflammatory bowel disease (IBD) is one of the chronic gastrointestinal diseases with increasing risk of colon cancer development in the future. Apoptosis and inflammation play an important role in the etiology of this disease. MicroRNAs are associated with etiology of different diseases including IBD. In this study, we aimed to explore the role of miR-135a in the etiology of colitis in murine model of DSS-induced colitis.

**Results:**

The results showed that expression of miR-135a in colonic cells was suppressed and up-regulating miR-135a inhibited apoptosis and inflammation of colonic epithelial cells. Additionally, Hif1α was identified as the target gene of miR-135a which promoted apoptosis and inflammation as knockdown of Hif1α led to the suppression of both apoptosis and inflammation.

**Conclusions:**

Overexpression of miR-135a might be beneficial in IBD due to its anti-apoptosis and anti-inflammation effects in vitro.

## Background

Inflammatory bowel disease (IBD) is a chronic condition which is characterized by inflammatory damage to small intestine and colon; the main IBD types include ulcerative colitis (UC) and Crohn’s disease (CD) [1]. The disease etiology is multifactorial and often occurs in genetically susceptible individuals due to aberrant inflammatory response to intestinal microbes. Besides the genetic hypothesis, environmental factors also play an important role in the etiology and progression of the disease [2].

Since World War II, the incidence of IBD is increasing worldwide. The most common reason cited is increased meat consumption due to which there is an increased intake of animal protein that is associated with IBD. IBD, especially UC (the risk is minimal with CD) is associated with increased risk of colon cancer [1, 2].

MicroRNAs (miRNAs) are single stranded noncoding RNA strands consisting of approximately 18–24 nucleotides; miRNAs are mainly responsible in post-transcriptional gene regulation. miRNAs bind the complementary 3′ untranslated regions (UTRs) of the messenger RNAs (mRNAs). The principal biological functions of miRNAs include alteration or restoration of gene function to facilitate adoption to physiological and pathological environment [3]. miRNAs are associated with several diseases including inflammatory diseases and cancers [3–5].

miRNAs are already known to play an important role in regulation of immune and inflammatory responses both in physiological and pathological (disease) conditions. Common inflammatory diseases include chronic obstructive pulmonary disease (COPD), alcoholic, drug-induced, and inflammatory liver injury, and etc [6, 7]. Similarly, several studies have also described the different expression patterns (genetic polymorphism) of different miRNAs (including miR-135a) in IBD patients and the potential role of different miRNAs in progression to colon cancer in these patients [5, 8, 9].

miR-135a is associated with number of diseases including cancer [10, 11], cardiovascular diseases [12], acute lung injury [13], Alzheimer’s disease [14], CD and UC [15]. Nowadays, most investigations focused on the role of miR-135a in cancers. The role of miR-135a is rather controversial as miR-135a is upregulated in hepatocellular carcinoma promoting metastasis [16]; similarly in bladder cancer miR-135a expression is upregulated [17]. However, in case of malignant glioma [11], epithelial ovarian cancer [18], and in lung cancer [19] miR-135a acts as a tumor suppressor. Iborra et al. reported that miR-135a was down-regulated in serum in CD and UC patients, implying that miR-135a might be an important regulator in inflammatory bowel diseases [15].

One of the most widely used animal models for colitis is murine model of dextran sodium sulfate (DSS), a chemical with anticoagulant properties. DSS is water soluble, negatively charged sulfated polysaccharide with a molecular weight ranging from 5 to 1400 kDa. It is not established the mechanism by which DSS induces colitis; the most probable cause is due to damage to the epithelial monolayer lining because of proinflammatory contents of the intestine like bacteria. The murine model of DSS was widely used in research purpose of inducting colitis because of its simplicity, reproducibility, rapidity and controllability of model development [20].

Hypoxia is associated with several diseases namely, hematological, cardiovascular, pulmonary and inflammatory bowel diseases. One of the key regulators of hypoxia is hypoxia inducible factor 1 (Hif1), which plays a complex role in inducing apoptosis or acting as an anti-apoptotic factor [21]. In this study, we have explored the role of miR-135a in DSS-induced murine model of colitis.

## Methods

### DSS Mouse Model of Colitis

Mice were fed with 4% (wt/vol) DSS (MW 36–50 KDa, MP Biomedical LLC, Solon, OH) dissolved in sterile distilled water (vehicle control) ad libitum for the duration of the experiment (days 0–8). Mouse weight was recorded as the initial weight before administration with DSS. After administration with DSS, the body weight of mice was recorded daily, and also recorded whether mice had the following symptoms: hematochezia, diarrhea, bloody diarrhea and weight loss. In order to confirm whether the mouse colitis model was successfully induced, the serum samples and colonic tissue samples were collected from the mice. Histopathologic examination was performed and relative miR-135a expression was detected. All animal work was approved by the Institutional Animal Care and Use Committee of Huaihe Hospital of Henan University and the local Experimental Ethics Committee.

### Histopathological analyses of mouse colon tissue

Colon segments were fixed in 10% neutral-buffered formalin, embedded in paraffin, sectioned at 5 μm, and stained with hematoxylin and eosin (H&E) for histopathological detection of severity of inflammation, extent of injury and crypt damage.

### TUNEL assay

TUNEL (deoxynucleotidyl transferase–mediated deoxyuridine triphosphate) (Beyotime, China) assay using paraffin-embedded tissues was conducted as manufacturer’s instructions. Slides were observed under fluorescent microscopy (Nikon Eclipse 80i, Japan).

### In situ *hybridization*

In situ hybridization was performed with 5′-locked digoxigeninlabeled LNA™ miR-135a probe complementary to mouse mature miR-135a and LNA™ U6 snRNA as positive control (Exiqon, Vedbaek, Denmark). Briefly, colon tissues were deparaffinized and deproteinized with protease K for 15 min at 37 °C. Slides were then washed twice with PBS and dehydrate with ethanol. Hybridization was performed at 37 °C for 28 h, followed by blocking with 0.3% BSA in PBS for 30 min. The probe-target complex was detected immunologically by incubating with a digoxigenin Ab conjugated to alkaline phosphatase acting on the chromogen NBT/5-bromo-4-chloro-3-indolyl phosphate (Sigma) for 16 h. Slides were counterstained with nuclear fast red, examined and photographed (Nikon Eclipse 80i, Japan).

### Colonic epithelial cell isolation and incubation

Colonic epithelial cells were isolated. Briefly, the colon was removed, everted, incubated for 30 min at 37 °C in a Ca-free Krebs-Henseleit buffer containing 2.5 mM dithiothreitol, 5 mM EDTA, and antibiotics (2.5 μg/ml amphotericin, 100 μg/ml kanamycin monosulfate, 250 U/ml penicillin G, and 250 μg/ml streptomycin sulfate). The epithelium was subsequently forced from the connective tissue with a pressurized stream of Krebs-Henseleit buffer containing 5 mM dithiothreitol and 0.025% bovine serum albumin. Epithelial cells were washed and resuspended in the buffer solution.

### Cell transfection assay

miR-135a overexpresison vector was constructed using pcDNA3. The recombinant vector pcDNA3 and miR-135a were constructed and after screening and identifying the recombinant, it was transfected into colonic cells. Antisense oligonucleotides (ASO)-miR-135a and its corresponding negative control (NC), i.e. ASO-NC, were all purchased from Genepharma (Shanghai, China). The siRNA of Hif1α (5’-AGGAAGAACTATGAACATAAA-3′) and the corresponding negative control were designed and synthesized by GenePharma. They were referred as to siRNA-Hif1α and siRNA-control. The cell transfection was performed using Lipofectamine 3000 (Invitrogen Life Technologies, Carlsbad, CA, USA) following the manufacturer’s protocol. After 48 h of transfection, cells were collected for forthcoming analyses.

### Enzyme-Linked Immunosorbent Assay (ELISA)

Culture supernatant was collected and the productions of inflammatory factors were determined by specific ELISA kits. Quantitative determination of IL-8, IL-6, and TNF-α was performed by following the manufacturer’s recommendations (R&D Systems, Minneapolis, MN). The lower detection limits were 31.2 pg/ml for IL-8, 9.4 pg/ml for IL-6, and 15.6 pg/ml for TNF-α.

### Western blotting

For western blotting, cells were lysed in RIPA buffer (50 mM Tris-HCl (pH 8.0), 150 mM NaCl, 1% NP-40, 0.5% sodium deoxycholate, and 0.1% SDS) with protease inhibitor cocktail (Roche, Basle, Switzerland) on ice for 30 min and cleared by centrifugation. 20 μg lysates were boiled at 100 °C for 5 min in 4 × SDS loading buffer and the samples were separated by SDS–PAGE, and transferred to a PVDF membrane. The membranes were blocked in 5% non-fat milk blocking buffer (Thermo Scientific, Shanghai, China) and then incubated overnight at 4 °C with primary antibody and detected by immunoblotting analysis with the indicated antibodies using Immobilon Western Chemiluminescent HRP Substrate (Millipore, Massachusetts, America). Band intensities were quantified by densitometric analyses using NIH ImageJ. All the experiments were performed at least three times and the most representative results were shown.

### RNA isolation and quantitative real-time PCR (qRT-PCR) analysis

Total RNA was extracted from cells using TRIzol reagent (Invitrogen) according to the manufacturer’s instructions. For miR-200a detection, cDNA was synthesized by Taqman MicroRNA Reverse Transcription Kit and PCR was performed by using Taqman Universal Master Mix II (Applied Biosystems, Foster City, CA, USA). For Hif1α detection, the cDNA was synthesized with PrimeScript 1st Strand cDNA Synthesis Kit (Takara, Japan) and the expression of Hif1α gene encoding was quantified by qPCR with SYBR Green Real time PCR Master Mix (TOYOBO, QPK-201). Amplification of cDNA was performed on an Applied Biosystems 7900HT Fast Real-Time PCR System. Cycling parameters were 95 °C for 1 min and then 40 cycles of annealed at 60 °C for 15 s, extended at 72 °C for 1 min. U6 was used as the endogenous control for miR-135a expression. β-actin was used as the endogenous control for Hif1α expressions. The relative expression was calculated using (2^−ΔΔCt^) method. The primer sequences were:

miR-135a, forward, 5’-ACACTCCAGCTGGGTATGGCTTTTTATTCCT-3’, and reverse, 5’-GGTGTCGTGGAGTCGGCAA-3’;

Hif1α, forward, 5’-ACTGCCACCACTGATGAATCAAAAACAG-3′, and reverse, 5′- TTCCATTTTTCGCTTCCTCTGAGCATTC-3′.

### Flow cytometry assay

Cell apoptosis was examined by flow cytometry through Annexin V-FITC/Propidium Iodide (PI) double staining assay. After different treatment, colonic epithelial cells were harvested, washed with cold phosphate buffered saline (PBS) and suspended in binding buffer containing 10 mM HEPES/NaOH (pH 7.4), 140 mM NaCl, 2.5 mM CaCl_2_ at a concentration of 10^6^ cells/mL. Then the cells were stained with Annexin V-FITC and PI followed and analyzed on the flow cytometer to evaluate the percentage of apoptotic cells. Experiment was repeated three times and data were analyzed with Cell Quest Software.

### 3’UTR luciferase reporter assay

3’UTR luciferase reporter plasmids and either miR-135a mimics or ASO-miR-135a were cotransfected in colonic epithelial cells. Cells were harvested at 48 h after transfection and luciferase activity was assayed with Dual-Luciferase Reporter Assay System on a luminometer (Berthold, LB9507, Germany) according to the manufacturer’s protocol (Promega). All experiments were performed in triplicate. The relative ratio of Renilla luciferase activity to Firefly luciferase activity was calculated for each well.

### Statistical analysis

All data are expressed as mean ± standard deviation and statistical analysis of the data was performed using SPSS 16.0. Statistically significant differences were assessed by one-way analysis of variance (ANOVA) or student’s t-test. *p < 0.05* was considered to be statistically significant.

## Results

### Expression of miR-135a in DSS-induced colitis

**Fig. 1:**
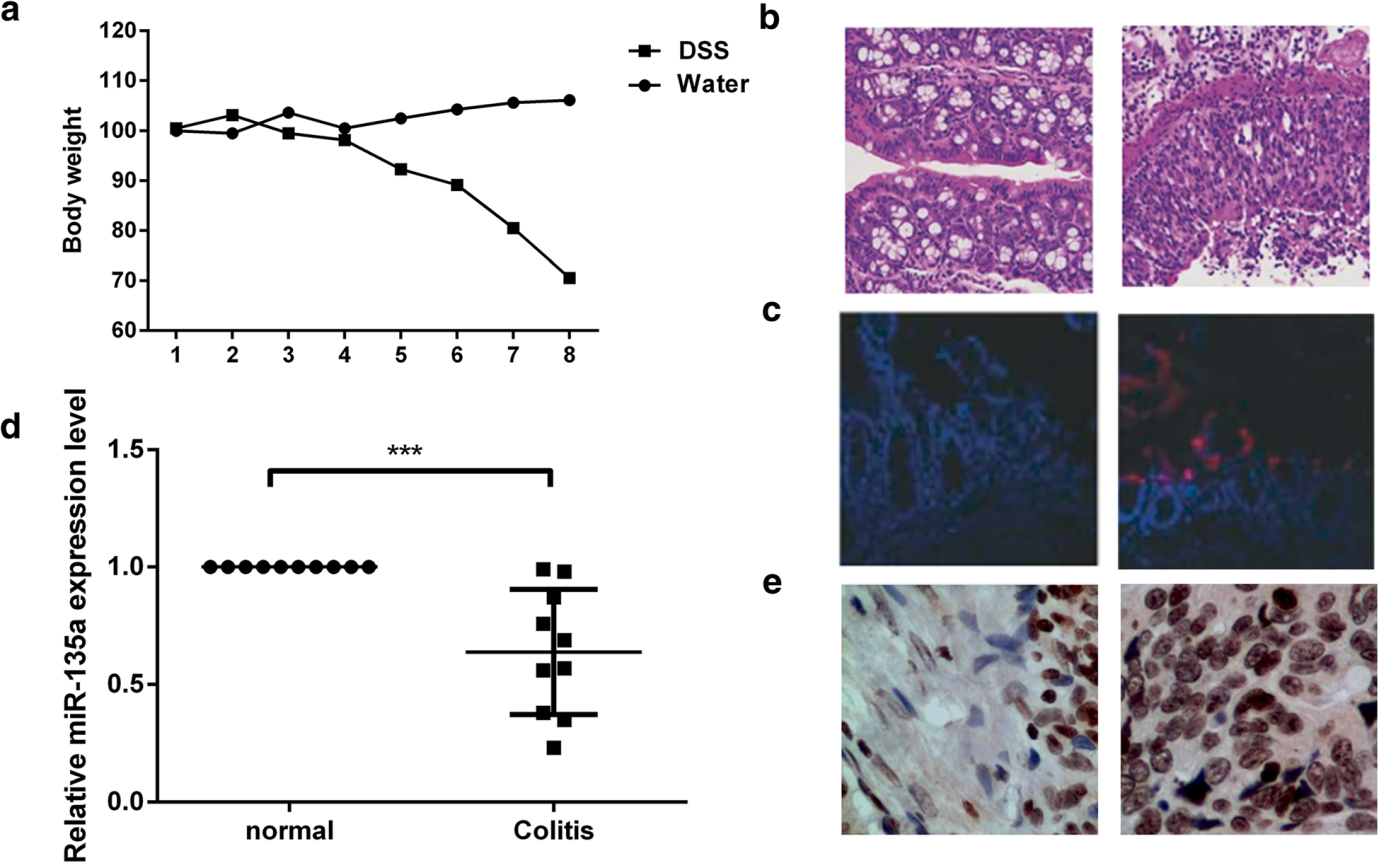
DSS caused symptoms of inflammation on mouse model and miR-135a was over-expressed in DSS-induced colitis. To determine severity of inflammation, **a** body weight was evaluated; **b** H&E staining of tissue and **c** TUNEL assay were conducted. To determine expression of miR-135a, **d** qRT-PCR and **e** in situ hybridization were conducted. miR-135a: microRNA-135a, DSS-induced colitis: Dextran sulfate sodium-induced colitis; H&E staining, hematoxylin and eosin staining; TUNEL assay, terminal transferase uridyl nick end labelling assay; qRT-PCR, quantitative reverse transcription-PCR. ^***^p < 0.001

Our experimental results revealed that the mice was presented with symptoms of diarrhea and significant weight loss after 8 days of administration of 4% DSS. After 8 days, the loss of body weight was about 30% compared to control group mice (Fig. 1a), indicating that DSS treatment resulted in a significant weight reduction. The pathological examination of tissue sections with H&E staining confirmed that the colonic epithelium and mucosal tissue of the mice were severely damaged and accompanied by a large number of inflammatory cell infiltration after 8 days of administration of 4% DSS (Fig. 1b). To investigate the consequence in cell apoptosis, we performed a TUNEL assay. After induction of colitis, mice model displayed a significantly higher number of apoptotic epithelial cells compared with normal mice (Fig. 1c). At the same time, we measured the expression level of miR-135a in mice by qRT-PCR and in situ hybridization (Fig. 1d and e). Consistently, miR-135a was significant down-regulated in colon of DSS-treated mice relative to control mice (*p* < 0.001).

### The functional role of miR-135a in DSS-induced murine colitis model

**Fig. 2:**
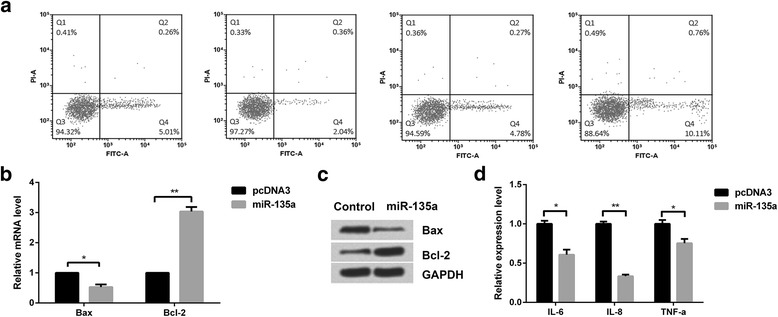
Role of miR-135a in apoptosis and inflammation of colonic epithelial cells. **a** Apoptotic cells were analyzed by flow cytometer. The expression levels of apoptosis-related proteins were determined by **b** qRT-PCR and **c** Western blot. **d** The expression levels of inflammatory cytokines were analyzed by ELISA. ELISA, enzyme-linked immunosorbent assay; miR-135a: microRNA-135a; ASO-miR-135a, antisense oligonucleotides-miR-135a. ^*^p < 0.05, ^**^p < 0.01

Apoptosis is one of the important reasons for initiation of inflammatory colitis. In order to explore the role of miR-135a in colitis, we performed flow cytometer analysis (Fig. 2a) to detect the effect of miR-135a on apoptosis of colonic epithelial cells. Next, we performed qRT-PCR and western blot to detect the expressions of apoptosis-related genes (Fig. 2b and c). The results showed that miR-135a could significantly inhibit the expression of Bax, the apoptosis-promoting factor, and promote the expression of Bcl-2, the anti-apoptotic factor. At the same time, we determined the levels of TNF-α, IL-8, and IL-6 (measured by ELISA), and found that miR-135a significantly inhibited the levels of IL-6 (*p < 0.05*), IL-8 (*p < 0.01*), and TNF-α (*p < 0.05*) (Fig. 2d).

### Identification of Hif1α as target gene of miR-135a

**Fig. 3:**
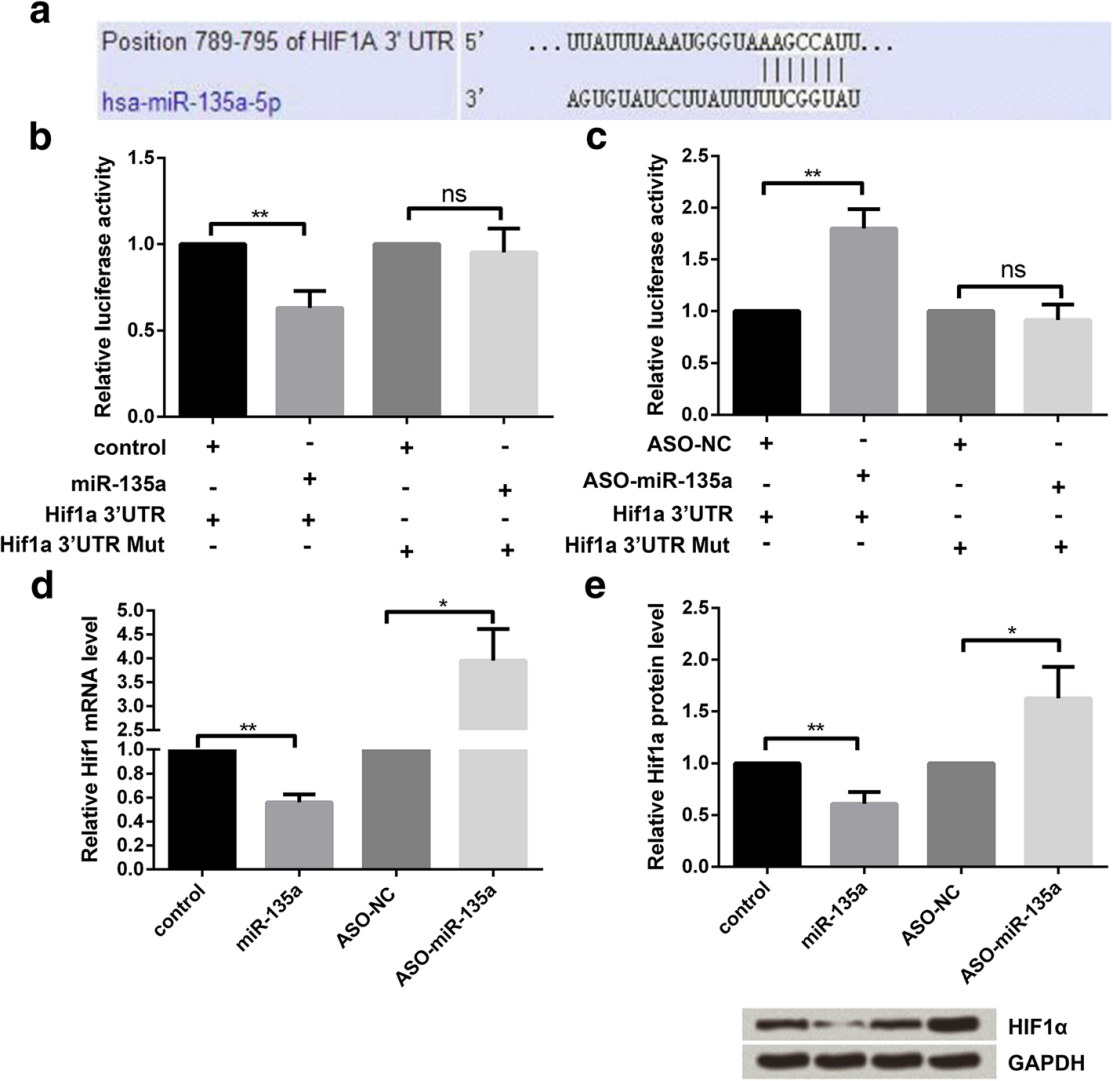
Hif1α was a direct target gene of miR-135a. **a** The predicted miR-135a binding sites on Hif1α. **b** and **c** Luciferase activity in cells cotransfected with miR-135a (or ASO-miR-135a) and luciferase reporters containing Hif1α-wild type or Hif1α-mutant type vector. The expression of Hif1α was regulated by miR-135a, according to **d** qRT-PCR analysis and **e** Western blot. miR-135a: microRNA-135a, Hif1α: Hypoxia-inducible factor 1-alpha. ns *p > 0.05,* ^*^p < 0.05, ^**^p < 0.01

We first predicted the candidate target gene by using TargetScan, and then confirmed that Hif1α was a target gene of miR-135a (Fig. 3a) and was also negatively regulated by miR-135a (confirmed by luciferase reporter assay, qRT-PCR, and western blot), (Fig. 3b-e).

### Knockdown of Hif1α inhibited apoptosis and inflammatory response in DSS-induced murine colitis model

**Fig. 4:**
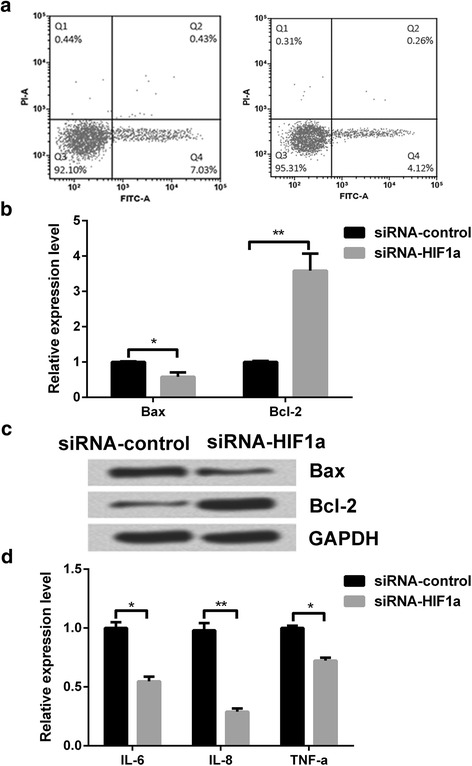
Effects of knockdown of Hif1α on apoptosis and inflammatory response of colonic epithelial cells. **a** Apoptotic cells were analyzed by flow cytometer. The expression levels of apoptosis-related proteins were determined by **b** qRT-PCR and **c** Western blot. **d** The expression levels of inflammatory cytokines were analyzed by ELISA. ^*^p < 0.05, ^**^p < 0.01

Knockdown of Hif1α led to the suppression of apoptosis and inflammation in the DSS-induced murine colitis model (Fig. 4a). Western blot analysis revealed significant fall (*p < 0.05*) in Bax, a pro-apoptic factor and significant increase (*p < 0.01*) in the level of Bcl-2, anti-apoptic factor, (Fig. 4b and c) similarly to those seen in cells overexpressing miR-135a. Again, similar to the effects of miR-135a, knockdown of Hif1α led to suppression of expressions of inflammatory mediators namely, IL-6 (*p < 0.05*), IL-8 (*p < 0.01*), and TNF-α (*p < 0.05*) compared to control group of cells (Fig. 4d).

## Discussion

Inflammatory bowel disease, including ulcerative colitis and Crohn’s disease, is a troublesome disease because of the symptoms [1, 2]. Although currently available treatment modalities, such as drugs and surgery, can effectively improve the quality of life in the patients, the risk of dysplasia and its progression into colon cancer especially in the patients suffering from ulcerative colitis cannot be eliminated completely [2].

miRNAs are widely researched as non-coding RNA molecules [3]. Few miRNAs act as tumor suppressors while some promote tumorigenesis. miR-135a plays a rather contradictory role as sometimes it promote tumorigenesis and sometime suppresses it [11, 16–19]. In colon cancer, miR-135a is known to facilitate progression of the disease [22]. Importantly, miR-135a was found to be reduced in serum in a study of CD and UC patients [15]. In this study, we tried to explore the role of miR-135a in DSS-induced murine model of colitis.

The expression of miR-135a was decreased in the mice with DSS-induced colitis and also it was seen that apoptosis was increased in mice compared to control group of mice. However, overexpression of miR-135a in the colonic cells led to suppression of apoptosis and inflammation. We also found that knockdown of the target gene of miR-135a, Hif1α, led to suppression of both apoptosis and inflammation. Therefore, it can be suggested that miR-135a exerts its anti-apoptotic and anti-inflammatory actions via suppressing the expression of Hif1α. Other studies have already mentioned the anti-apoptotic function of miR-135a. Similar to our findings, miR-135a suppressed apoptosis of HL-1 cells in diabetic mice [23], promoted proliferation, induced migration and tenogenic differentiation of tendon stem/progenitor cells [24], and ameliorated allergen-induced inflammation in allergic rhinitis [25].

Hif1α can act either as a pro-apoptotic or anti-apoptotic factor depending on the cell type. Studies have established beneficial role in targeting Hif1α in cancer therapy [26]. In our study, we have also found the anti-apoptotic effect of Hif1α down-regulation.

DSS-induced murine model of colitis is one of the widely used animal models to study the pathogenesis, molecular changes and to evaluate drug action on IBD including ulcerative colitis. IBD is a multifactorial disease; the most popular hypothesis implies that triggering of apoptosis and dysregulation of inflammatory process in the colonic mucosa might lead to IBD in genetically susceptible individuals [1, 2].

In this study, we have demonstrated that the expression of miR-135a was suppressed in the DSS-induced murine model of colitis and increased expression of miR-135a was associated with suppression of apoptosis and inflammation. Hence, it can be suggested that suppression of miR-135a function leads to the development of DSS-induced colitis (as apoptosis and inflammation are the two most important factors in pathogenesis of colitis).

## Conclusions

miR-135a was down-regulated in DSS-induced colitis in mice. Up-regulating miR-135a inhibited apoptosis and inflammatory response in colonic epithelial cells. Besides, Hif1α directly targeted by miR-135a participated in the function of miR-135a in colonic epithelial cells. Thus, it can be concluded that increased expression of miR-135a and further down-regulated Hif1α is beneficial in protecting the colonic mucosa from inflammatory colitis.

## Abbreviations

CD: Crohn’s disease
COPD: chronic obstructive pulmonary disease
DSS: dextran sodium sulfate
Hif1: hypoxia inducible factor 1
IBD: inflammatory bowel disease
PBS: phosphate buffered saline
UC: ulcerative colitis
UTRs: untranslated regions
